# Sitting Sideways Causes Different Femoral-Tibial Rotations in Each Knee

**DOI:** 10.7759/cureus.59678

**Published:** 2024-05-05

**Authors:** Kenichi Kono, Shoji Konda, Takaharu Yamazaki, Shuji Taketomi, Masashi Tamaki, Hiroshi Inui, Sakae Tanaka, Tetsuya Tomita

**Affiliations:** 1 Department of Orthopaedic Surgery, The University of Tokyo, Bunkyo, JPN; 2 Department of Health and Sport Sciences, Osaka University, Suita, JPN; 3 Department of Information Systems, Saitama Institute of Technology, Fukaya, JPN; 4 Department of Orthopaedic Surgery, Graduate School of Medicine, Osaka University, Osaka, JPN; 5 Department of Orthopaedic Surgery, Saitama Medical University, Kawagoe, JPN; 6 Department of Medical Sciences, Morinomiya University of Medical Sciences, Osaka, JPN

**Keywords:** axial rotation, normal knee, kinematics, kneeling, sitting sideways

## Abstract

Purpose

According to a previous study, asymmetrical kneeling, such as sitting sideways, does not exhibit asymmetrical movements. Rotational analyses of each femur and tibia help explain why rotational knee kinematics while sitting sideways do not exhibit asymmetrical movement. We aimed to assess the rotation of the femur and tibia in normal knees while sitting sideways.

Methods

Each volunteer sat sideways under fluoroscopy. Two-dimensional and three-dimensional registration techniques were used. After evaluating the femoral rotation angle relative to the tibia at each flexion angle, the femoral and tibial sole rotation angles at each flexion angle were compared between the ipsilateral and contralateral knees.

Results

While sitting sideways, both knees showed femoral external rotation relative to the tibia with flexion. In the ipsilateral knees, the femurs exhibited an external rotation of 26.3 ± 8.0°, from 110° to 150° of flexion. Conversely, the tibia exhibited an external rotation of 12.2 ± 7.8°, from 110° to 150° of flexion. From 110° to 150° of flexion, femoral external rotation was significantly larger than tibial external rotation. In the contralateral knees, the femurs exhibited an internal rotation of 23.8 ± 6.3°, from 110° to 150° of flexion (110°, p < 0.001; 120°, p < 0.001; 130°, p < 0.001; 140°, p < 0.001; and 150°, p < 0.001). Contrastingly, the tibia exhibited an internal rotation of 30.4 ± 8.8°, from 110° to 150° of flexion, which was significantly larger than femoral internal rotation (110°, p = 0.002; 120°, p < 0.001; 130°, p < 0.001; 140°, p < 0.001; and 150°, p < 0.001).

Conclusions

Although bilateral knees exhibited femoral external rotation relative to the tibia while sitting sideways, the ipsilateral and contralateral knees showed femoral and tibial sole rotations in opposite directions. In particular, the contralateral knees might show a strained movement because both femurs and tibias exhibited internal rotation with flexion. Patients who have undergone guided-motion total knee arthroplasty (TKA) or medial-pivot TKAs might be advised to avoid sitting sideways.

## Introduction

With the diversification of activities of daily living (ADL), people desire various knee motions, such as walking, stair activity, squatting, kneeling, and sitting cross-legged. In addition, previous studies have reported that the kinematics of normal knees differ depending on the activities [1-7]. During walking, a lateral pivot motion is generally observed [5]. By contrast, during squatting and kneeling, a medial pivot motion has been observed with flexion [3,4]. Furthermore, while sitting cross-legged, a lateral pivot motion followed by a medial pivot motion has been observed [4]. Therefore, the evaluation of each activity is important.

During sitting activities, such as sitting on the floor, praying, holding tea ceremonies, and gardening, a kneeling motion is mandatory. In addition, there are several types of kneeling styles, such as seiza-sitting and sitting sideways. Knee kinematics during seiza-sitting has indicated a sharp femoral external rotation with a medial pivot [4]. While sitting sideways, the ipsilateral knees exhibit external femoral rotation and a medial pivot motion with flexion, whereas the contralateral knees exhibit internal femoral rotation and a lateral pivot motion with flexion. However, a previous study demonstrated that bilateral knees show femoral external rotation and the contralateral knees do not show lateral pivot motion. In other words, normal knees exhibit external femoral rotation during asymmetrical kneeling. Moreover, lateral pivot motion was not observed, even in the contralateral knees, during asymmetrical kneeling. Even in asymmetrical kneeling, the knees do not exhibit asymmetrical movement [8]. To investigate why the rotational knee kinematics while sitting sideways do not exhibit asymmetrical movement, rotational analyses of each femur and tibia are essential.

In this study, we aimed to examine the rotation of the femur and tibia in normal knees while sitting sideways. We hypothesized that the sole rotation would differ between the femur and tibia.

This article was previously posted to the medRxiv preprint server on April 27, 2020.

## Materials and methods

Twelve knees from six volunteers were examined. Approval from the institutional review board was obtained through documentation, and all volunteers provided written informed consent to participate in the study. All values are expressed as mean ± SD.

During the fluoroscopy, each volunteer sat sideways at a natural pace [8]. They practiced the motion several times for a few minutes before recording. The right and left knee motions were recorded separately. The sequential motion was captured as a series of digital radiography images (1024 × 1024 × 12 bits/pixel, 7.5-Hz serial spot images as a DICOM file) using a 17-inch (43 cm) flat panel detector system. Furthermore, all images were processed using dynamic-range compression, thereby enabling edge-enhanced images. To estimate the spatial position and orientation of the knee, a two-dimensional/three-dimensional (2D/3D) registration technique was employed [4,9].

Moreover, 3D bone models were created using CT and used as computer-aided design (CAD) models. The estimation accuracy for the relative motion between 3D bone models was ≤ 1° in rotation and ≤ 1 mm in translation [4].

**Figure 1:**
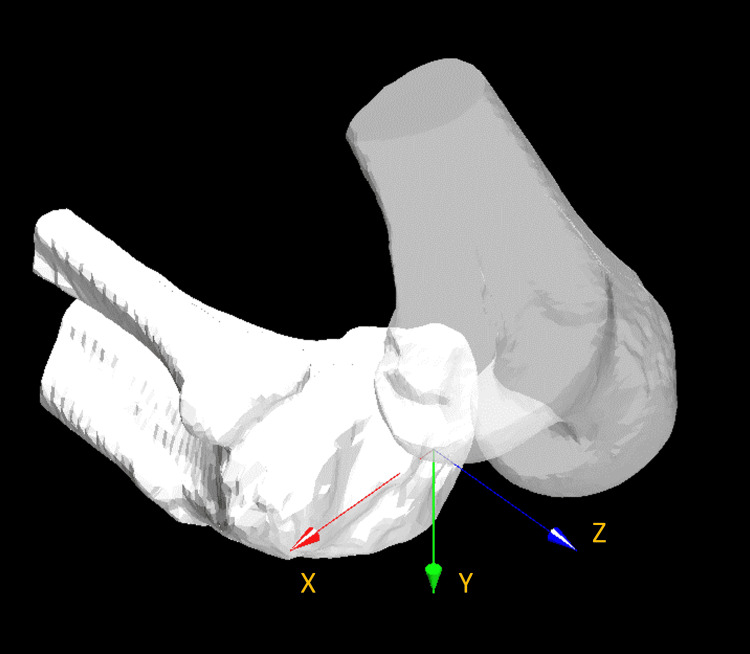
The global coordinate system. A vertical line from the tibial origin was established as the Y-axis. A line passing through the centre of medial and lateral eminences and the ankle centre was projected onto the plane perpendicular to the Y-axis, and the projection was established along the Z-axis. A line perpendicular to both Y- and Z-axes was denoted as the X-axis.

A local coordinate system was produced for the bone model, as described in a previous study [10]. Knee rotations were described using the joint rotational conventions of Grood ES and Suntay WJ [11]. The femoral rotation angle relative to the tibia was evaluated [4,8]. External and internal rotations were denoted as positive and negative, respectively. The rotational angles of the femur and tibia were then calculated. In the global coordinate system, the vertical line from the tibial origin was established as the Y-axis. The line that passed through the centers of the medial and lateral eminences and the ankle center was projected onto the plane perpendicular to the Y-axis. This projection was established along the Z-axis. The line perpendicular to both the Y- and Z-axes was established as the X-axis (Figure 1).

**Figure 2:**
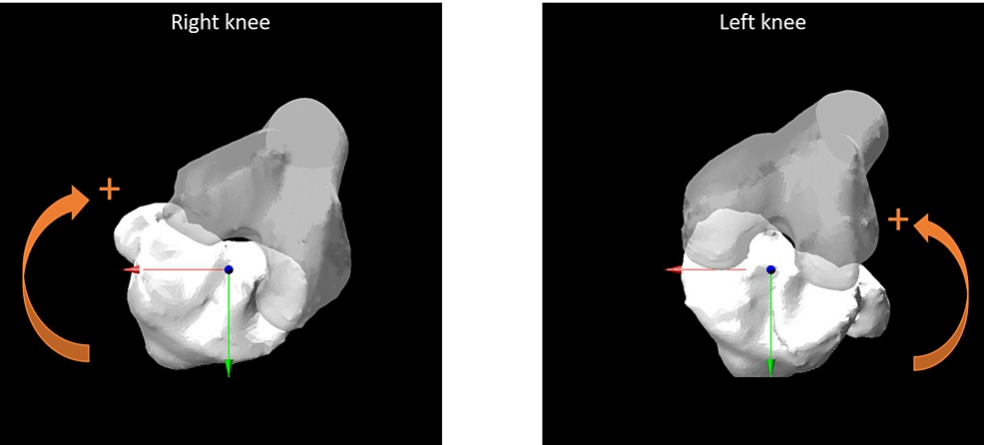
Femoral and tibial sole rotation. In the AP view, clockwise and anticlockwise rotations of the right and left knees, respectively, were defined as the external rotation and represented as positive. AP: Anteroposterior.

The femoral and tibial rotations relative to the global coordinate system were defined as the femoral and tibial sole rotations, respectively. In the AP view, clockwise and anticlockwise rotations of the right and left knees, respectively, were defined as external rotations and represented as positive (Figure 2).

The femoral and tibial sole rotation angles at each flexion angle were compared between the ipsilateral and contralateral knees.

Statistical analyses

Results were analyzed using SPSS version 24 (IBM Corp., Armonk, NY, USA) with repeated-measures ANOVA and post hoc pairwise comparisons (Bonferroni test). Statistical significance was set at p < 0.05. Moreover, a power analysis using EZR [12] indicated that 11 knees would be required to achieve an alpha of 0.05 and a power of 0.8.

## Results

Volunteer’s demographics

All volunteers were Japanese men, whose mean age at the time of examination was 37.3 ± 7.6 years, mean height was 169.9 ± 5.2 cm, mean weight was 64.2 ± 5.2 kg, and mean body mass index was 22.2 ± 1.1 kg/m² (Table 1) [8]. The volunteers did not have any previous relevant surgical history or trauma that could affect their range of movement.

**Table 1:** Volunteer’s demographics.

Demographic	Mean ± SD
Age (years)	37.3 ± 7.6
Body height (cm)	169.9 ± 5.2
Body weight (kg)	64.2 ± 5.2
BMI (kg/m^2^)	22.2 ± 1.1

Femoral flexion and rotation relative to the tibia

While sitting sideways, the ipsilateral knees were gradually flexed from 98.4 ± 6.8° to 150.8 ± 4.5°, and the contralateral knees were gradually flexed from 101.7 ± 6.2° to 155.2 ± 4.8°.

**Figure 3:**
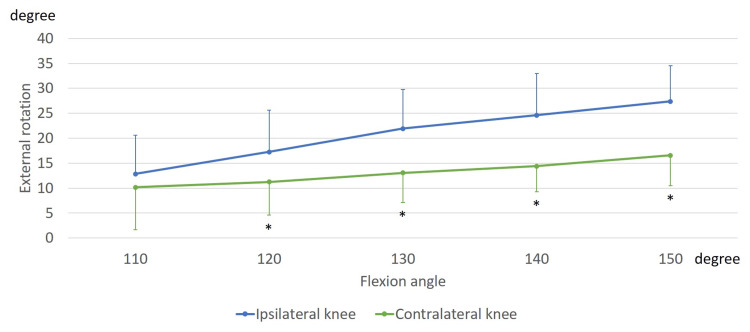
Rotation angle while sitting sideways. The markers indicate femoral rotation relative to the tibia. *, significant differences between the ipsilateral and contralateral knees (p < 0.05).

In the ipsilateral knees, the femurs exhibited an external rotation of 13.7 ± 3.5° relative to the tibia, from 110° to 150° of flexion. In the contralateral knees, the femurs exhibited an external rotation of 5.8 ± 6.8° relative to the tibia, from 110° to 150° of flexion (Figure 3). From 120° to 150° of flexion, the femoral external rotation in contralateral knees was significantly smaller than that in ipsilateral knees (120°, p = 0.008; 130°, p = 0.001; 140°, p < 0.001; and 150°, p < 0.001).

Femoral and tibial sole rotation angles

**Figure 4:**
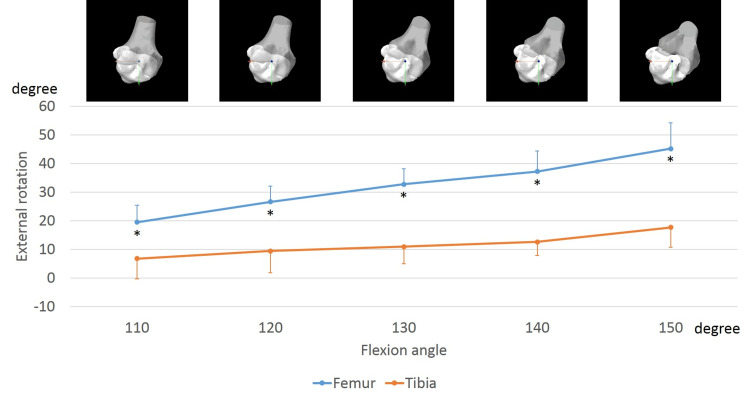
Sole rotation of the femur and tibia in the ipsilateral knees while sitting sideways. The markers indicate femoral and tibial rotation relative to the global coordinate system. The anteroposterior view of each flexion angle is shown. *, significant differences between the femur and tibia (p < 0.05).

In the ipsilateral knees, the femurs exhibited an external rotation of 26.3 ± 8.0° from 110° to 150° of flexion. Conversely, the tibia exhibited an external rotation of 12.2 ± 7.8°, from 110° to 150° of flexion (Figure 4). From 110° to 150° of flexion, the femoral external rotation was significantly larger than the tibial external rotation (110°, p < 0.001; 120°, p < 0.001; 130°, p < 0.001; 140°, p < 0.001; and 150°, p < 0.001).

**Figure 5:**
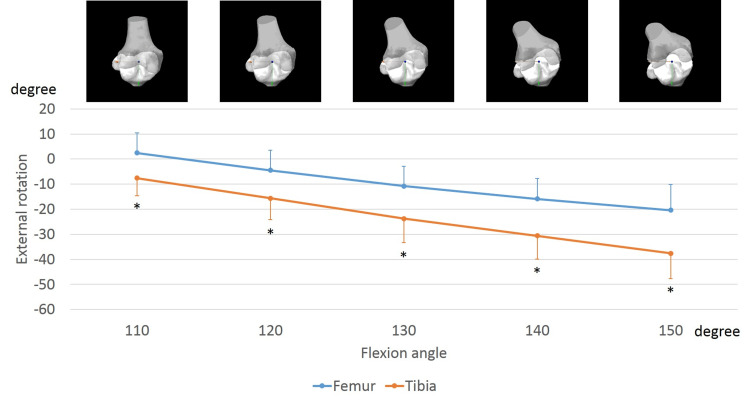
Sole rotation of the femur and tibia in the contralateral knees while sitting sideways. The markers indicate femoral and tibial rotation relative to the global coordinate system. The anteroposterior view of each flexion angle is shown. *, significant differences between the femur and tibia (p < 0.05).

In the contralateral knees, the femurs exhibited an internal rotation of 23.8 ± 6.3° from 110° to 150° of flexion. In contrast, the tibia exhibited an internal rotation of 30.4 ± 8.8°, from 110° to 150° of flexion (Figure 5). This was significantly larger than the femoral internal rotation (110°, p = 0.002; 120°, p < 0.001; 130°, p < 0.001; 140°, p < 0.001; and 150°, p < 0.001).

## Discussion

The most important finding of the current study was that although both the ipsilateral and contralateral knees exhibited femoral external rotation relative to the tibia with flexion, the femoral and tibial sole rotations differed between the ipsilateral and contralateral knees. In the ipsilateral knees, both the femur and tibia exhibited external rotation with flexion. Furthermore, femoral external rotation was significantly greater than tibial external rotation during flexion. This difference in external rotation was attributed to the femur's external rotation relative to the tibia. By contrast, in the contralateral knees, both the femur and tibia exhibited internal rotation with flexion. Additionally, tibial internal rotation was significantly larger than femoral internal rotation during flexion. This difference in internal rotation was attributed to the external rotation of the femur relative to the tibia. Although both knees exhibited femoral external rotation relative to the tibia while sitting sideways, the rotational mechanism differed between the ipsilateral and contralateral knees.

With the recent diversification of ADL, patients who undergo TKA also desire to sit with a deep knee bend. Several studies have demonstrated that knees after TKA exhibit femoral external rotation [13-15]. Niki Y et al. reported that seiza-sitting after TKA appeared safe in terms of component dislocation [14]. Furthermore, Nakamura S et al. has reported that ball-and-socket joint articulation enables patients to kneel safely without dislocation [15]. In contrast, medial pivot TKA was recently introduced based on normal knee kinematics, with reportedly good or excellent clinical outcomes [16-21]. Patients who undergo medial pivot TKA are advised to avoid asymmetrical sitting, such as sitting sideways, because the contralateral knee is capable of exhibiting a lateral pivot motion with femoral internal rotation. A previous study demonstrated that both knees showed femoral external rotation; additionally, the contralateral knees did not show lateral pivot motion [8]. This suggests that normal knees exhibit femoral external rotation during asymmetrical kneeling. Moreover, lateral pivot motion may not be observed in the contralateral knees during asymmetrical kneeling. The rotation of the ipsilateral knees while sitting sideways is relatively similar to that of normal knees during symmetrical kneeling [3,4]. By contrast, the rotational movement of the contralateral knees while sitting sideways was minimal. In addition, the femur and tibia exhibited internal rotation with flexion in the contralateral knees. Therefore, although patients who underwent conventional TKAs might not need to avoid sitting sideways, those who underwent guided-motion TKAs or medial-pivot TKAs might want to avoid kinematic conflict while sitting sideways.

This study had some limitations. First, it analyzed the knee joint kinematics of only healthy Japanese men. The knee kinematics of women, other races, and patients with osteoarthritis may differ. Second, in this study, the right and left knee motions were recorded separately because it was impossible to record bilateral knees in a flat panel. Therefore, our findings cannot be applied to simultaneous knee motion while sitting sideways.

## Conclusions

Although bilateral knees exhibited femoral external rotation relative to the tibia while sitting sideways, the femoral and tibial sole rotations were in opposite directions in the ipsilateral and contralateral knees. In particular, the contralateral knees might show a strained movement because both femurs and tibias exhibited an internal rotation with flexion. The patients who underwent guided-motion TKAs or medial-pivot TKAs might want to avoid kinematic conflict while sitting sideways.
